# Case Report: Dermoscopic features of oral lichen planus - the evolution of mucoscopy

**DOI:** 10.12688/f1000research.14134.2

**Published:** 2018-03-27

**Authors:** Sidharth Sonthalia, Sangeeta Varma, Abhijeet Kumar Jha, Deepak Jakhar, Feroze Kaliyadan

**Affiliations:** 1 Skinnocence: The Skin Clinic & Research Centre, C-2246, Sushant Lok-1, Block-C, Gurugram, 122009, India; 2 Department of Dermatology, Kalyani Hospital & Twachapal Skin Clinic, Gurugram-122016, Haryana, India; 3 Department of Dermatology & STD, Patna Medical College & Hospital (PMCH), Ashok Rajpath, Patna-400008, Bihar, India; 4 Department of Dermatology & STD, Hindu Rao Hospital, Sabji Mandi, New Delhi, 110007, India; 5 Department of Dermatology, College of Medicine, King Faisal University, Hofuf, Saudi Arabia

**Keywords:** dermoscopy, mucoscopy, lichen planus, oral, mucosal, leukoplakia, veil-like, speckled-pearly erosion, dotted, linear, curvilinear, vessels, clods, brown

## Abstract

Dermoscopy, a non-invasive technique for cutaneous diagnosis is being increasingly studied in various disorders of the skin, nails and scalp. However, it has been under-utilized for the diagnosis and characterization of mucosal disorders. The dermoscopic characterization of cutaneous lichen planus and its variants has been well documented with Wickham’s striae constituting the hallmark of the condition. However, the dermoscopic features of oral lichen planus with hand-held or videodermoscopy remain to be elucidated. We present the case of a young adult man who presented with asymptomatic white lacy lesions over a bluish-black background over the tongue, patchy hyperpigmentation of the buccal mucosae and gingivae, and longitudinal melanonychia involving some nails. History of intake of any drugs preceding the lesions, smoking, chewing of betel nut and dental implants was negative. Family history was non-contributory. There were no cutaneous lesions suggestive of lichen planus. Mucoscopy (dermoscopy of the mucosa, oral in this case) and onychoscopy were done followed by biopsy from the tongue that confirmed the diagnosis of lichen planus. Oral mucoscopy of the tongue revealed a tri-colored pattern with structureless veil-like grey-white areas (modified Wickham’s striae), well-demarcated red glossy erosions, and violaceous-to-brown clods. Additionally, vascular pattern of dotted and linear to curved vessels along the borders of leukoplakia-like areas and erosions were observed. Onychoscopy confirmed lichen planus-associated melanonychia. Dermoscopy also proved useful in conveniently ruling out other disorders typified by mucosal and nail pigmentation such as Laugier Hunziker syndrome and drug-induced changes. Although direct oral microscopy has been used in defining features of oral lichen planus, to the best of our knowledge this case is the first report on mucoscopy or dermoscopy of oral lichen planus

## Introduction

Dermoscopy has unleashed opportunities of exploring structures and features of the skin invisible to the unaided eye. Inflammoscopy, i.e. dermoscopy of inflammatory dermatoses has sufficiently advanced to the point of facilitating dermoscopic differentiation between plaque psoriasis, eczema and pityriasis rosea ^1^.

Wickham striae (WS) characterized by white crossing streaks are the dermoscopic hallmark of cutaneous LP ^1– 4^. A background of dull red color, and vessels of mixed morphology (dotted and linear) represent additional dermoscopic findings of LP ^1, 5^. There is paucity of data on dermoscopy of mucosal, especially oral lichen planus (OLP), which is encountered in more than one-third cases of cutaneous LP, with an estimated global prevalence of 0.5–2.2% ^6– 8^.

## Case details

**Figure 1.**
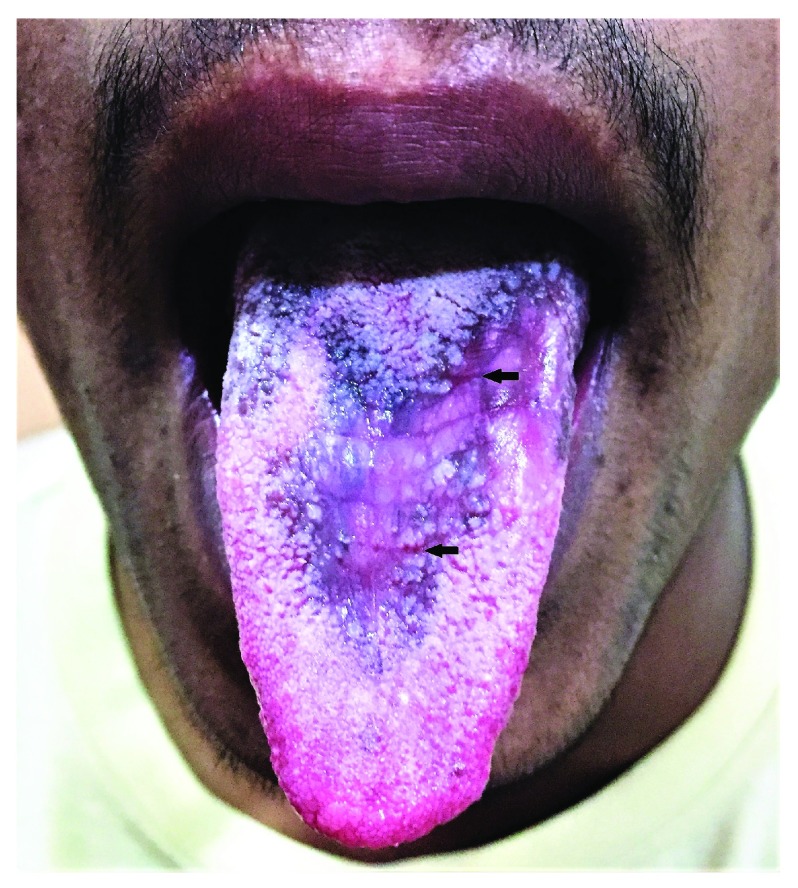
Dorsum of the tongue revealing violaceous to dark grey discoloration and blunted papillae in the centre with few tiny bright red erosions (black arrow) and interspersed white reticular lesions.

**Figure 2.**
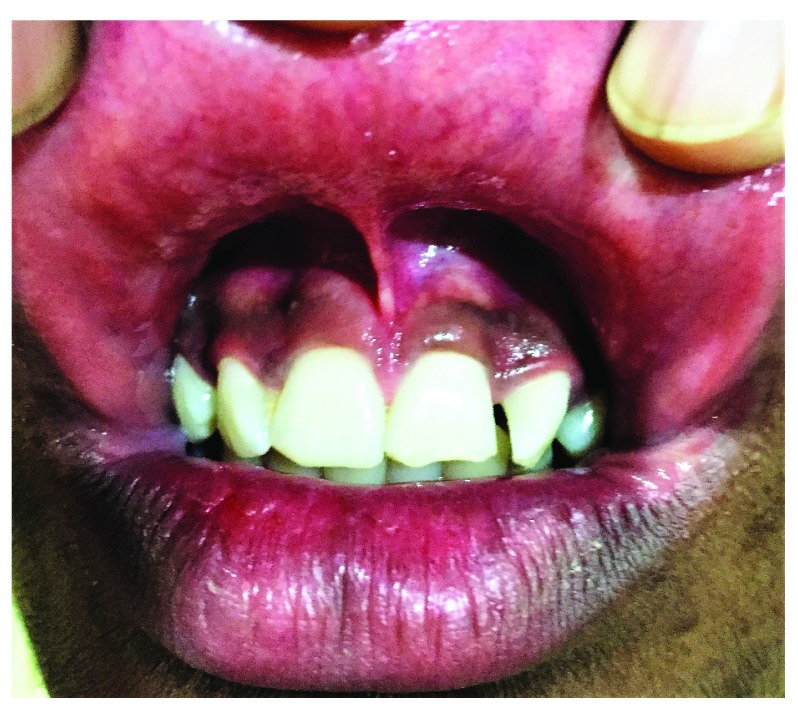
Mild desquamative gingivitis with gingival hyperpigmentation.

**Figure 3.**
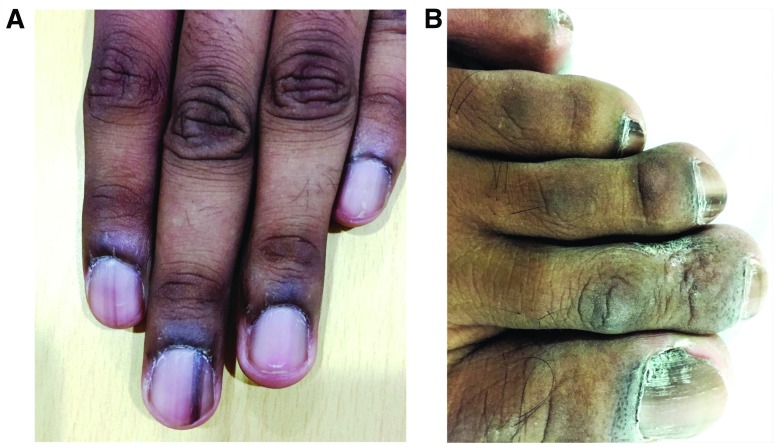
Longitudinal melanonychia of multiple ( **a**) fingers of the left hand, and ( **b**) toes of the right foot. Pigmentation of the proximal and lateral nail folds is conspicuous.

A 19-year-old Indian gentleman was evaluated for asymptomatic patchy pigmentation over multiple finger and toe nails, the tongue, and buccal cavity, noticed eighteen months back. There was no history of preceding trauma, drug intake, soreness of mouth, or dental procedures or amalgam filling. He denied addictions like smoking or chewing of betel nut or tobacco. Current and past medical history were unremarkable. There was no history of parental consanguinity, familial nail pigmentation or any familial pigmentary disorder. Examination of oral mucosa revealed poor oral hygiene. The dorsum of the tongue revealed violaceous to dark grey discoloration extending onto the ventral surface, interspersed with white reticular lesions and focal tiny bright red erosions ( Figure 1). Buccal mucosae revealed brown colored macules with focal presence of white reticular lesions. Lingual papillae projections appeared blunted in the discolored central area. Although mild desquamative gingivitis with gingival hyperpigmentation were appreciable, the lips were spared with no visible freckling ( Figure 2). Examination of nails revealed longitudinal melanonychia of multiple fingers and toe nails ( Figure 3). Relevant hematological and biochemical investigations ruled out hepatitis, dyslipidemia, diabetes and thyroid disorder.

## Dermoscopic features

**Figure 4.**
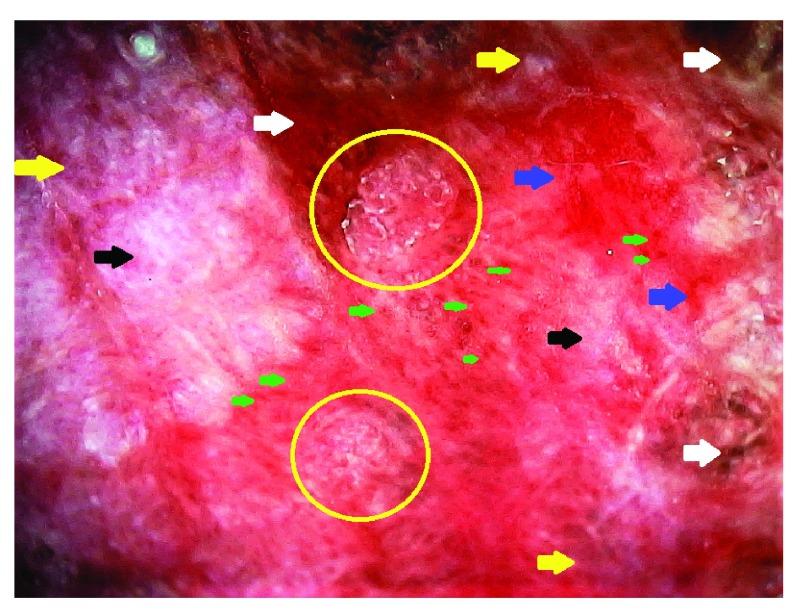
Dermoscopic image from dorsum of the tongue displaying tri-color pattern constituted by 1) Modified Wickham’s striae appearing like structureless veil-like grey-white (black arrows) to bluish-white areas (yellow arrows), which have also been called leukoplakia-like areas (LLAs) and speckled pearly appearance (yellow circles); 2) bright red slightly depressed areas suggestive of erosions (blue arrows), and 3) interspersed violaceous-to-brown clods (white arrows) suggestive of pigment incontinence. Scattered dotted as well as linear to curvilinear vessels (green arrows) present along the borders of the junction of ‘white’ and ‘red’ areas. (Escope Videodermoscope, 20×, polarized mode). The blunting of lingual papillae is better appreciated on comparison with Figure 5.

**Figure 5.**
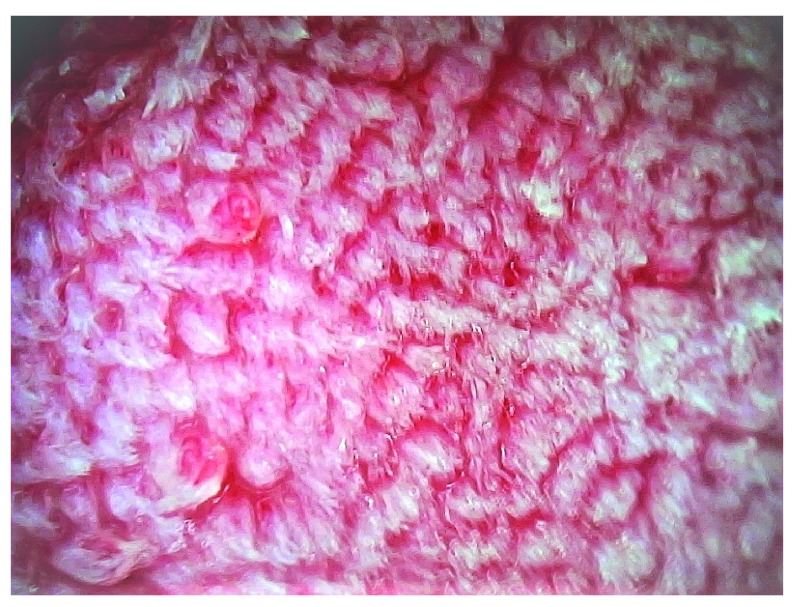
Dermoscopic image from dorsum of the tongue adjacent to the lesion showing normal pattern of fungiform lingual papillae in contrast to the blunting of papillae seen in Figure 4. (Escope Videodermoscope, 20×, polarized mode).

**Figure 6.**
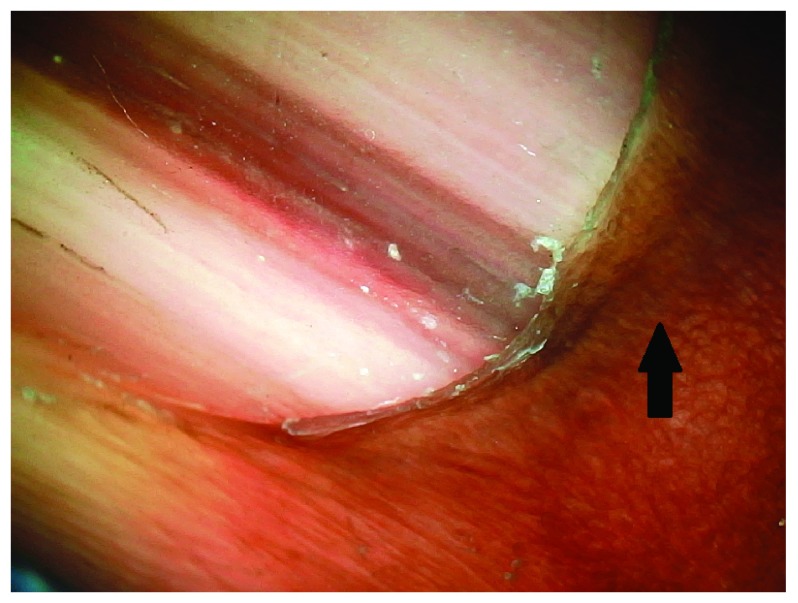
Dermoscopic image of a finger nail showing uniformly pigmented parallel linear band of pigmentation with pseudo-Hutchinson sign (black arrow). (Escope Videodermoscope, 20×, polarized mode).

Video dermoscopy (EScope; polarized mode, ×20) of the dorsum of the tongue revealed blunting of papillae ( Figure 4), in contrast to the preserved papillary pattern observed in the peripheral portion ( Figure 5). The affected area displayed a tri-color pattern constituted by – 1) structureless veil-like grey-white to bluish-white areas, 2) bright red slightly depressed areas, and 3) interspersed violaceous-to-brown clods. A few foci of specked-pearly white structures were also observed ( Figure 4). Dotted and linear to curvilinear vessels were visible at the junction of the white and red areas. Dermoscopy from the surrounding normal-appearing areas of the tongue dorsum revealed the typical fungiform lingual papillae ( Figure 5). Dermoscopy from buccal mucosa only revealed diffusely spread violaceous clods. Onychoscopy revealed multiple 3–4 mm wide uniformly pigmented parallel linear bands of pigmentation with pseudo-Hutchinson sign ( Figure 6).

## Investigations and diagnosis

**Figure 7.**
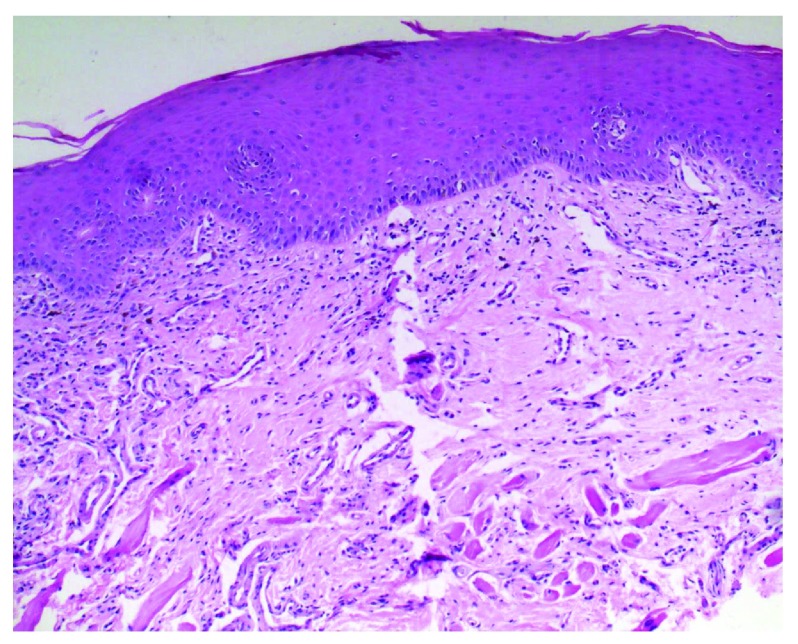
Histopathological picture of biopsy from tongue dorsum with typical features of mucosal lichen planus (H & E, 100x).

A 10% KOH smear from the oral mucosa was negative for candidiasis. Histopathology revealed irregular acanthosis, basal layer vacuolization, necrotic keratinocytes, moderately dense interface dermatitis .and pigment incontinence ( Figure 7). A final diagnosis of erosive oral lichen planus was made.

## Discussion

The dermoscopic features of cutaneous LP are typified by the presence of a dull red background, white crossing streaks of WS (multiple patterns), and mixed pattern of dotted and linear vessels at the periphery of the lesions ^1– 5^. OLP may occur in isolation, or in association with cutaneous and/or nail LP. Buccal mucosa and tongue are most commonly affected, followed by gums and labial mucosa ^9^. In contrast to the well documented dermoscopic features of cutaneous LP, lichen planus pigmentosus, nail lichen planus and lichen planopilaris ^10^, the dermoscopic characterization of OLP is almost non-extant. To the best of our knowledge, there is a single case report of dermoscopy of LP involving the lower lip ^11^. Drogoszewska *et al*. in their study, employed direct oral microscopy, a non-invasive diagnostic technique based on the principles of dermoscopy and colposcopy, to describe the *in vivo* picture of erosive OLP. The purpose of this study was to evaluate the role of the technique as a guide to selecting optimal biopsy site to reveal dysplastic changes ^12^. In their study, Drogoszewska *et al*. described the typical ‘direct microscopic’ picture of erosive OLP as bi-colored consisting of planar to minimally elevated, dull white, hyperkeratotic leukoplakia-like areas (LLA) lesions, and well-demarcated, bright red and glossy erosions with a smooth moist surface, present adjacent to the LLAs ^12^. Further, they reported subepithelial capillaries to be invisible within the lesions. In the current case, three different colors and patterns were observed by video dermoscopy of OLP – veil-like structureless greyish-white areas, bright red well-demarcated erosions, and interspersed violaceous-to-brown colored clods. The latter are suggestive of sub-epithelial pigment incontinence. Thus a tri-colored, pattern was observed.

The pattern and appearance of WS was different in oral mucosal LP compared to the pattern typical of cutaneous LP. In cutaneous LP, WS most commonly present as white streaks in a reticular pattern, although other patterns have been reported including circular, radial streaming, linear, globular, veil-like, leaf venation, and starry sky/white dots ^10, 13, 14^. In the current case, WS presented as – veil-like structureless grey-white to bluish white areas, and specked-pearly pattern in few foci. It is interesting to note, that such modified appearance of WS has also been reported at another mucosal site, the vulva. In the dermoscopic evaluation of 10 women with vulvar LP, Borghi *et al*. reported that WS in more than half the patients gave a similar veil-like structureless grey-white to blue-white appearance ^15^. They also observed white homogenous areas in 50% patients ^15^.

In our experience, LP involving the cutaneous aspect of the lip displays the typical WS, whereas the mucosal aspect shows WS resembling LLAs. Dotted and linear to curvilinear vessels were visible at the junction of the white and red areas, akin to the vascular pattern observed in dermoscopy of cutaneous LP. The fourth feature from the tongue lesion was blunting of lingual papillae. This feature may depend on the morphological sub-type of OLP.

The onychoscopic findings of uniform-colored 3–4 mm broad bands of longitudinal melanonychia and the pseudo-Hutchinson’s sign stemming from hyperpigmentation of the nail bed and matrix reflecting through the transparent nail folds may be seen in LP, with other common reported causes being racial pigmentation, Laugier-Hunziker syndrome (LHS), and drug-induced melanonychia ^16^.

## Conclusion

We suggest that a tri-colored pattern constituted by modified WS with a veil-like grey-white to bluish-white structureless morphology (or LLAs) and focal speckled-pearly appearance, red erosions, and violaceous-to-brown clods, in addition to dotted and linear to curved vessels along the junction of LLAs and erosions are characteristic of OLP. Last but not the least, akin to the evolution of other sub-specialties of dermoscopy (trichoscopy, inflammoscopy, entomodermoscopy, onychoscopy etc.), mucoscopy needs to be explored more to extend the versatility of dermoscopy for diagnosis of mucosal disorders.

## Data availability

The data referenced by this article are under copyright with the following copyright statement: Copyright: © 2018 Sonthalia S et al.

Data associated with the article are available under the terms of the Creative Commons Zero "No rights reserved" data waiver (CC0 1.0 Public domain dedication). 

*No data is associated with this article.*

## Consent

Written informed consent for publication of the clinical details and clinical images was obtained from the patient himself.
