# Supplementary material for: Case Report: Dermoscopic features of oral lichen planus - the evolution of mucoscopy
Source: F1000Res. 2018 Mar 27;7:284. Originally published 2018 Mar 6. [Version 2] doi: 10.12688/f1000research.14134.2 (PMC5861519; doi:10.12688/f1000research.14134.2)
Supplement: Peer review history

# Peer review: Referee response for version 2

**DOI:** 10.5256/f1000research.15654.r32507

**Published:** 2018-03-28

**Authors:** Sukesh MS

**Affiliations:** 1 Cosmetic Dermatology & Hair Restoration Centre, Bangalore, Karnataka, India

I have read this submission. I believe that I have an appropriate level of expertise to confirm that it is of an acceptable scientific standard.

# Peer review: Referee response for version 1

**DOI:** 10.5256/f1000research.15374.r31603

**Published:** 2018-03-16

**Authors:** Sukesh MS

**Affiliations:** 1 Cosmetic Dermatology & Hair Restoration Centre, Bangalore, Karnataka, India

This case report enumerates the dermoscopic findings of oral lichen planus, which is described less in literature. The images, descriptions of each dermoscopic finding, histopathological correlation and discussion is aptly written. Wickham striae seen as veil-like structureless grey-white to bluish white areas, and specked-pearly pattern are highlighting dermoscopic features described here , for oral lichen planus. These findings need to be validated on larger studies.

 Short comings - 

 1] Mention of how the videodermoscope was used / modified to examine the buccal mucosa either though a description or through an image , would have been more informative 

 2] Correction of description of image 4  - 'Yellow areas' to 'yellow arrows' ; needs to be done

I have read this submission. I believe that I have an appropriate level of expertise to confirm that it is of an acceptable scientific standard.

# Peer review: Referee response for version 1

**DOI:** 10.5256/f1000research.15374.r31604

**Published:** 2018-03-15

**Authors:** Balachandra S. Ankad

**Affiliations:** 1 Department of Dermatology, S Nijalingappa Medical College, Bagalkot, Karnataka , India; 2 Rajiv Gandhi University of Health Sciences, Bengaluru, Karnataka, India

**Dear Authors,**

1. Very good manuscript.
2. In introduction, mention full form of LP.
3. Figure number 4; bluish-white areas are labeled by yellow arrows but mentioned as yellow areas. Kindly change it.

**Comments**

1. It is a good manuscript describing and characterizing dermoscopic patterns in oral lichen planus.
2. Authors have detailed each dermoscopic pattern seen in the case.
3. Discussion is authentic and covered the necessary aspects of it.

**Strengths of manuscript**

1. Very good dermoscopic image.
2. Authors have correlated dermoscopic and histopathologic changes very well.
3. Description of dermoscopic image is appropriate.

**Weakness of manuscript**

1. It is a report of single case, hence needs more validation of dermoscopic findings.

I have read this submission. I believe that I have an appropriate level of expertise to confirm that it is of an acceptable scientific standard.
